# Polymorphisms of Tumor Necrosis Factor-***α***, Transforming Growth Factor-***β***, and Interleukin-10 in Asthma Associated with Olive Pollen Sensitization

**DOI:** 10.1155/2014/276345

**Published:** 2014-12-21

**Authors:** Blanca Cárdaba, David Calzada, Selene Baos, Miriam Aguerri, Joaquín Quiralte, Carlos Lahoz

**Affiliations:** ^1^Immunology Department, IIS-Fundación Jiménez Díaz, UAM, Avenida Reyes Católicos No. 2, 28040 Madrid, Spain; ^2^CIBER de Enfermedades Respiratorias, CIBERES, Instituto de Salud Carlos III, Monforte de Lemos No. 3-5, 28029 Madrid, Spain; ^3^Allergy Department, Hospital Universitario Vírgen del Rocío, Avenida Manuel Siurot, s/n, 41013 Seville, Spain

## Abstract

Sensitization to specific olive pollen-allergens (Ole e 2 and 10) has been correlated with a clinical pattern of asthma. This study analyzes the association between several polymorphims of* TNFA* (*G-308A*,* C-857T*, and* C-1031T*),* IL10* (*C-571A* and* A-1117G*), and* TGFB* (*C-509-T*) and these sensitizations. These polymorphisms were genotyped by allelic discrimination, in olive pollen-allergic patients (phenotyped for specific Ole e 2 and 10 sensitizations) and healthy controls. Levels of serum-soluble cytokines were correlated with specific genotypes and clinical phenotypes. The results showed that heterozygous* TGFB C-509T* genotype, besides having the lowest sera TGF- levels, was significantly increased in olive pollen-allergic patients compared with controls. According specific sensitizations,* CC* genotype of* IL10 C*-*571A* could be a protective factor for Ole e 2 sensitization and mainly for asthmatic Ole e 2 sensitized patients compared with asthmatic non-Ole e 2 sensitized patients (OR: 0.26, *P* = 0.008). In contrast, heterozygous* CA* genotype was increased in Ole e 2 asthmatic subjects compared to asthmatic non-Ole e 2 sensitized patients. Lastly, heterozygous* TNFA G-308A* genotype was associated with Ole e 10 sensitization (OR: 2.5, *P* = 0.04). In conclusion, these results suggest a role of TGF-*β*1 in olive-pollen sensitization and TNF-*α* and IL-10 genotypes in the asthma induced by specific olive-pollen allergens.

## 1. Introduction

Allergic diseases are adverse reactions of the immune system against otherwise harmless substances. The reason why exposure to common environmental antigens induces allergic responses in some people and not others remains undetermined. Atopy and asthma have a complex genetic background, and multiple genes can contribute to their development through main effect, gene-gene, and gene-environmental interactions.

Allergen-specific CD4+ helper T-cell (TH) generation is the initial event leading to the development of allergic disease. TH2 subtypes are pivotal to the inflammatory cascade through the production of IL-4, IL-5, IL-13, and IL-9. TH1 cells (secreting mainly IL-12 and IFN-*γ*) may contribute to the chronicity and effector phase of these diseases. Several genetic polymorphisms of these genes have been studied in relation to allergic diseases and asthma [1–3].

However, other important molecules have been associated with phenotypes of asthma, including tumor necrosis factor- (TNF-) *α*. This proinflammatory cytokine has been found in increased concentrations in asthmatic airways and the inhalation of TNF-*α* has been shown to cause airway hyperresponsiveness and increased sputum neutrophil counts in healthy volunteers [4]. TNF-*α* is a member of the* TNF* gene superfamily located within the human major histocompatibility complex on chromosome 6p, linked to atopic asthma in several studies [5, 6]. Several polymorphisms have been reported in the promoter region of the* TNFA* gene [7], with* TNF*-308G>A being the polymorphism most widely studied in relation to this disease.

In addition to the inflammatory events, regulatory T-cell dysfunction is associated with development of complex genetic conditions such as atopy and asthma. Peripheral T-cell tolerance is characterized by functional inactivation of the cells in contact with the antigen, which in turn eliminates both proliferate response and cytokine secretion [8, 9]. In humans, several T-cell subtypes with an immune-suppressive function, generically named regulatory T cells (Treg), have been extensively studied [10, 11]. The main role of all these cell subsets is to maintain the integrity of the body by avoiding excessive immune responses that may result in harmful immune pathology, as well as preserve a state of tolerance to innocuous substances [12]. IL-10 and TGF-*β* secreted by these Tregs play an important role in the immune regulatory response [13–15]. Several common polymorphisms have been identified in the promoter region of both cytokines, including −*1082G>A* and −*592C>A* for* IL10* and −*509C>T* for* TGFB* genes [7], some of these polymorphisms being associated with allergic diseases [16–19].

Olive tree pollen is one of the most important causes of respiratory allergy in the Mediterranean area.* Olea europaea* pollen induces mainly nasal and conjunctive symptoms, although it may also induce asthma exacerbations in areas with high levels of* O. europaea* pollen in the atmosphere. In Jaén, a region in southern Spain, there is a very high level of pollen (500 to 1000 grains/m^3^ during pollen season, with peaks of more than 5000 grains/m^3^) and a high prevalence of asthma [20].

To date, at least 20 proteins with allergenic activity have been described in olive pollen. Among them, Ole e 1 is the most frequent sensitizing allergen. Besides Ole e 1, twelve additional allergens have also been isolated and purified from* Olea europaea* pollen extract, some of which are major allergens in areas with high levels of pollen exposure, such as Ole e 2 and Ole e 10 [21, 22]. Previously our group described specific genetic and environmental factors associated with olive pollen allergy [23–27], as also a strong association between Ole e 10 and Ole e 2 specific sensitizations and bronchial asthma clinical phenotype, being these sensitizations restricted by different HLA class II antigens [22]. More recently we have described how, during the pollen season, olive pollen allergic patients showed a statistically significant decrease of TGF-*β* (regulatory cytokine). This result was consistent with a significant decrease in relative FOXP3 mRNA expression (marker of regulatory T-cell cytokines) and with the lower number of regulatory T cells, indicating a lack of regulatory mechanisms in olive pollen allergic subjects during the pollen season [28]. The subjects were selected from a region in southern Spain with particularly high pollen counts during the pollen season and a high prevalence of asthma.

Taking into account all of these previous results, we analyzed the role of 6 polymorphisms of genes previously associated with allergy and asthma, in a population of olive pollen allergic patients, with an extremely high incidence of asthma:* TNFA* (*G-308A, C-857T, *and* C-1031T*) as proinflammatory gene and* IL10* (*C-571-A *and* A-117G*) and* TGFB* (*C-509T*) as genes related to regulatory response.

## 2. Material and Methods

### 2.1. Subjects

We studied a population described previously [22]. One hundred forty-six unrelated patients were recruited at the Allergy Service of the “Complejo Hospitalario de Jaén.” All patients fulfilled the following criteria: seasonal rhinitis and/or asthma from April to June, a positive skin prick test for* O. europaea* pollen extract (ALK Abelló, Madrid, Spain), and no previous* O. europaea* immunotherapy.

Fifty healthy subjects from the same geographic area were recruited as a control group.

### 2.2. Clinical Assessment

Clinical assessment performed in these patients was described previously [22]. Patients recorded symptom scores, drug requirements, and peak expiratory flow rates (PEFR) every day from April to June (pollen season). Individual nasal and eye symptoms (sneezing, blockage, running, redness, and itching) and chest symptoms (breathlessness, wheezing, and tightness) were recorded on a scale of 0–3 (0 = no symptoms; 1 = mild symptoms; 2 = moderate symptoms; 3 = severe symptoms). The main clinical variable analyzed was defined as an asthma day. An asthma day fulfilled at least one of the following criteria: asthma symptoms score ≥2, morning PEF 20% lower than the mean morning PEF of the last 7 days before pollination, and daytime use of salbutamol twice or more compared with the mean use of the last 7 days before pollination.

### 2.3. Olive Pollen Allergens

Olive tree pollen and olive pollen allergens were isolated as previously was described [29, 30].

### 2.4. Total and Specific IgE Antibody Measurements

Total serum IgE levels and specific* O. europaea* extract IgE antibodies were determined by Pharmacia systems (IgE enzyme immunoassay and UNI-CAP, Uppsala, Sweden). Allergen-specific IgE antibody measurements (Ole e 1, Ole e 2, and Ole e 10) of allergic sera were performed by enzyme-linked immunoassay (ELISA) [22].

### 2.5. Skin Testing

All 146 patients and 50 controls were tested for olive pollen allergy by a skin prick test with* O. europaea* whole extract and olive pollen purified allergens, following the recommendations of the European Academy of Allergy and Clinical Immunology (EAACI) [31].

The patients were also tested with a panel of common aeroallergens, including* Dermatophagoides pteronyssinus, Blattella germanica*, dog and cat dander,* Alternaria alternata, Aspergillus fumigatus, Cladosporium herbarum, Lolium perenne, Cynodon dactylon, Cupressus sempervirens, Platanus acerifolia, Artemisia vulgaris, Chenopodium album, Salsola kali,* and* Parietaria judaica* (Abelló, Madrid, Spain).

### 2.6. Measurement of Soluble Cytokine Levels

TNF-*α* and IL-10 sera levels were analyzed by flow cytometry, using the BD Cytometric Bead Array (CBA), human TH1/TH2 Cytokine Kit (Becton Dickinson, BD, San Diego, CA). Flow cytometric analysis was performed using a FACSCalibur flow cytometer (BD Immunocytometry Systems, San Jose, CA). Data were gathered and analyzed by BD Cytometric Bead Array (CBA) Software. TGF-*β* sera levels were analyzed by a commercial ELISA (BD, San Diego, CA, USA).

### 2.7. Molecular Analysis

Genomic DNA was extracted from peripheral blood by using the salting out method.

Genomic DNA from all individuals was genotyped for 6 polymorphisms:* TNFA *(*G-308A, C-857T, *and* C-1031T)*,* IL10* (*C-571-A *and* A-1117G*), and* TGFB* (*C-509T*). Polymorphisms were analyzed according to the Applied Biosystems allelic discrimination assay-by-design protocol (AB-7500 Real Time PCR System). Single nucleotide polymorphisms (SNPs) in research and their identifier names, locations, alternative names, and dbSNP identification are shown in Table 1.

### 2.8. Statistical Analysis

Comparisons of phenotypic (olive pollen sensitized versus controls, asthmatic versus rhinitis) or genotypic frequencies between groups were measured by Fisher's exact test or by chi-square test.

Cochran and Mantel-Haenszel and 2-factor ANOVA tests were used for the statistical comparison of clinical parameters (qualitative and quantitative, resp.) between sensitized and nonsensitized groups. Multiple regression analysis was used to examine quantitative traits (total IgE, specific IgE to* O. europaea* whole extract, specific olive pollen allergens IgE, and soluble cytokines serum levels).

## 3. Results

### 3.1. Patients

Clinical features of patients included in the study were previously described [22]. A summary of the main characteristics is shown in Table 2.

The control group included 33 females (66%) and 17 males (34%), with a mean age of 37.6 years (range 23–58 years). These individuals were selected from the same geographical area and were free from any allergic symptoms. They were matched for gender and age. No relevant differences were found.

### 3.2. Olive-Allergen Sensitization

All the allergic subjects (*n* = 146) showed significant IgE antibody levels against pollen crude extract, but different frequencies of sensitization were observed for the purified* O. europaea *allergens (Table 2). Ole e 1, Ole e 2, and Ole e 10 are major allergens in our population (>50% of recognition frequency) [22].

### 3.3. Molecular Analysis

The genotype distribution of the 6 polymorphisms examined was in Hardy-Weinberg equilibrium.

Genotype and allelic frequency distribution in the patient and control groups and the genotype and allelic distribution according to asthma or rhinitis in our patient population are shown in Table 3.

The only statistically significant polymorphism associated with* O. europaea* allergy was related to the* TGFB-C509-T *polymorphism. We found a statistically significant increase in the phenotypic percentage of the* CT* heterozygous genotype in patients compared with controls (56.8% versus 40%, *P* = 0.03, odds ratio, OR, 1.97; 95% confidence interval, CI, 1.02–3.8) (Table 3). The sera of* CT* heterozygous patients showed the lowest levels of soluble TGF-*β* (10849.71 ± 12613 pg/mL) compared with* CC* (11526.6 ± 12613 pg/mL) and* TT* (15868.04 ± 10015 pg/mL) homozygous patients, but the differences were not statistically significant (data not shown).

When we analyzed the genotype distribution of the 6 polymorphisms according to the clinical phenotype of asthma or rhinitis, no relevant associations were found (Table 3).

However, the analysis according to specific Ole e 2 and Ole e 10 sensitizations showed several relevant results which are summarized in Table 4. The data shown are only the statistically significant results. Firstly, the study of 2 polymorphisms of* IL10* (*C-571-A *and* A-1117G*) in patients with Ole e 2 sensitization showed a statistically significant decrease of* CC* homozygous* IL10-571C>A *genotype compared with Ole e 2 nonsensitized patients (50% versus 70.5%, *P* = 0.029, OR, 0.41; 95% confidence interval, CI, 0.19–0.82). This protection was higher when patients with asthma and Ole e 2 sensitization were compared with Ole e 2 nonsensitized patients with asthma (49.4% versus 78.6%, *P* = 0.008, OR, 0.26; 95% CI, 0.09–0.72). This genotype showed intermediate levels of IL-10 cytokine (7.77 ± 5.4 pg/mL) compared with the other 2 genotypes (*AA* homozygous: 5.9 ± 6.1 pg/mL,* CA* heterozygous: 8.76 ± 5.3 pg/mL).

*CA* heterozygous* IL10-571C>A* genotype was increased in patients with asthma and Ole e 2 sensitization compared with Ole e 2 nonsensitized patients with asthma (45.7% versus 21.4%, *P* = 0.02, OR, 3.08; 95% CI, 1.13–8.4).

Secondly (Table 4), we have found a statistically significant increase of* GA* heterozygous* TNFA-308G>A* genotype in Ole e 10 sensitized patients compared with olive pollen allergic patients without sensitization to Ole e 10 allergen (25.3% versus 11.9%, *P* = 0.04, OR, 2.5; 95% CI, 1.02–6.1). This polymorphism showed intermediate levels of TNF-*α* cytokine (7.77 ± 5.4 pg/mL) compared with the other 2 genotypes (*GG* homozygous: 7.01 ± 4.8 pg/mL,* AA* homozygous: 9.3 ± 6.1 pg/mL).

## 4. Discussion

Sensitization to specific allergens is a complex response controlled by both genetic and environmental factors. Preliminary studies reported that the allergograms of olive-allergic patients living in areas with high level of pollen production (such as some regions as Andalusia in Spain) are notably different from those obtained in areas of low-level exposure (e.g., Madrid in the central region of the country) [32]. Besides, the level of each allergenic protein in the pollen grain could be a second parameter to be taken into consideration. In fact, a great variability in the allergen composition of* O. europaea* pollen has been described [33]. The reasons behind this variability are unknown, but they could be due to the existence of several genetic strains, climatic conditions, and specific culture techniques.

Previous works showed how, in areas with high levels of antigenic load, olive pollen allergic patients were sensitized to allergens that are “minor allergens” (frequency of recognition lower than 50%) in areas with low antigenic load [21, 22]. This sensitization (mainly related to Ole e 2 and Ole e 10) was associated with a worse clinical prognosis, with a very high presence of asthma and more days of symptomatology [22]. This population was analyzed for inflammatory cytokine polymorphisms (*IL-13, IL4RA, IL5,* and*β2ADR* genes) finding some relevant associations, mainly related to risk factor for olive pollen allergic sensitization [27].

Very recently we demonstrated how several immuneregulatory elements are decreased (TGF-*β* and FOXP3) in olive pollen allergic patients exposed to extremely high olive pollen antigenic load. [28].

With these previous results, the aim of this work was to determine whether some of the genes previously associated with asthma and immune regulation are important in these specific sensitizations. To do so, we decided to analyze the relationship between 6 genetic polymorphisms of* TNFA* (*G-308A, C-857T, *and* C-1031T*) as a proinflammatory gene and* IL-10* (*C-571-A *and* A-117G*) and* TGFB* (*C-509T*) as being related to regulatory response and specific sensitization to* O. europaea *pollen in a well-characterized Spanish population of patients with olive pollen allergy and a high prevalence of asthma (74.7%).

In spite of the small size of our control population, several preliminary conclusions can be drawn from our data. Firstly, we have shown that the* CT* genotype of* TGFB C-509T* was the only polymorphism (of the 6 analyzed) associated with the genetic regulation of* O. europaea* allergy (Table 3). TGF-*β* is a multifactorial cytokine that plays key roles in normal cellular processes and diseases, such as T-cell activation and proliferation, and may be essential in modulation of allergic airway inflammation and airway remodeling. The* TGF-β* gene is located on chromosome 19q and there are a number of polymorphisms within this gene that are believed to have a role in TGF-*β* expression [7].* TGFB* (*C-509T*) is presented within a proximal negative regulatory region, and the T allele has been associated with higher TGF-*β*1 plasma levels [34]. Due to the inconclusive results on the association of TGF-*β* polymorphisms and asthma, 3 years ago, a meta-analysis [35] was reported studying the role of TGF-*β* and asthma, based on 16-case control sets. The authors concluded that the C*-509T *polymorphism (carriers of the T allele) could be a risk factor for asthma, mainly in the Asian population and adults. This conclusion has been confirmed recently in other Asian populations [36]. Our results are in agreement with the association of* TT* homozygous and the highest levels of soluble TGF-*β*, but this genotype was infrequent in our population. However, the heterozygous* CT* genotype, which showed the lowest levels of TGF-*β*, was a risk factor for olive pollen allergy in our population. These data support our previous results in which we reported a TGF-*β* decrease in the sera of olive pollen allergic patients, in an independent cohort of subjects, selected from the same area [28]. All of these results reinforce our previous idea that olive pollen allergic patients have a decreased TGF-*β* dependent regulatory response, thereby pointing to the importance of evaluating, when feasible, the combination of levels and cytokine polymorphisms.

According to Ole e 2 and Ole e 10 specific sensitizations, we have found some interesting results (Table 4). Firstly, the* CC* homozygous* IL10-571C>A *genotype could be a protective factor for Ole e 2 sensitization and mainly for patients with asthma and Ole e 2 sensitization compared with asthmatic-patients nonsensitized to Ole e 2. This polymorphism showed intermediate levels of IL-10 cytokine (7.77 ± 5.4 pg/mL) compared with the other 2 polymorphisms (*AA* homozygous: 5.9 ± 6.1 pg/mL,* CA* heterozygous: 8.76 ± 5.3 pg/mL). In contrast, the* CA* heterozygous* IL10-571C>A *genotype was increased in patients with asthma and Ole e 2 sensitization compared with Ole e 2 nonsensitized patients with asthma (45.7% versus 21.4%, *P* = 0.02, OR, 3.08; 95% CI, 1.13–8.4).

A recent meta-analysis suggested that IL-10 promoter polymorphisms were associated with asthma risk [19]. This work revealed significant associations between −1082A/G and −592A/C polymorphisms and asthma. However, there was no significant association between −819T/C polymorphism and asthma risk. Other studies showed that a polymorphism at the −592 position of IL-10 is associated with its regulation of expression and recently the association of this polymorphism and immune-related diseases has been studied including type 2 diabetes with and without nephropathy, multiple sclerosis, and asthma [37].

Secondly, we have found a statistically significant increase of the* GA* heterozygous* TNFA-308G>A *genotype in Ole e 10 sensitized patients compared with olive pollen allergic patients without sensitization to the Ole e 10 allergen (25.3% versus 11.9%, *P* = 0.04, OR, 2.5; 95%, CI, 1.02–6.1) (Table 4). This genotype showed intermediate levels of the TNF-*α* cytokine (7.77 ± 5.4 pg/mL) compared with the other 2 genotypes (*GG *homozygous: 7.01 ± 4.8 pg/mL,* AA* homozygous: 9.3 ± 6.1 pg/mL).

The −308G/A polymorphism in the TNF-*α* gene has been extensively investigated for its association with asthma; however, the results of different studies have been inconsistent. Two years ago a meta-analysis was carried out to analyze the association between the −308G/A polymorphism of the TNF-*α* gene and asthma risk [38]. The results suggested that the TNF-*α*  − 308G/A polymorphism contributes to susceptibility to asthma and, specifically, significantly elevated risks of asthma were associated with A allele carriers in the atopic population but not in the nonatopic population. More recently [39], it has been described that the genetic polymorphisms of TGF-*β*1 and TNF-*α* are associated with asthma. This same study also described TGF-*β*1 as being a modulator of atopy, and both TGF-*β*1 and TNF-*α* were found to be elements that modulate clinical severity and airway obstruction in an additive manner.

Overall, our results suggest a role of TGF-*β*1 in olive pollen sensitization and TNF-*α* and IL-10 genotypes in the asthma induced by specific olive pollen allergens. Combined analysis of multiple genetic polymorphisms with different allergic phenotypes could be a useful tool for identifying profiles of risk or prevention of these disorders.

## Figures and Tables

**Table 1:** Main characteristics of the polymorphisms analyzed.

Gene	Polymorphism	Location	Alternative identifiers	dbSNP rs number^*^	Allele
*TNF*-*α*	G-308A	Promoter	*TNFA-*308G>A	rs1800629	G/A
	C-857T	Promoter	*TNFA*-857C>T	rs1799724	C/T
	T-1031C	Promoter	*TNFA*-1031T>C	rs1799964	T/C

*IL*-10	C-571A	Promoter	*IL-10571*C>A	rs1800872	C/A
	G-1117A	Promoter	*IL-101117*G>A	rs1800896	G/A

*TGF*-*β*	C-509-T	Promoter	*TGFB-509*C>T	rs1800469	C/T

*TNF-α*: tumor necrosis factor alpha chain, *IL-10*: interleukin-10, and *TGF-β*: transforming growth factor beta chain. ^*^ http://www.ncbi.nlm.nih.gov/SNP.

**Table 2:** Clinical features of the olive pollen allergic population.

Clinical features	
Sex (% female/male)	63/37
Mean age (years)	22.9 ± 7.1
Total IgE (IU/mL)	401.8 ± 502
*O. europaea* IgE (AU/mL)	36.9 ± 36
Atopic diseases	
Bronchial asthma	109 (74.7%)
Rhinitis	37 (25.3%)
Frequency of recognition (mean specific (OD/mL) sera levels)	
Ole e 1	91.7% (0.98 ± 0.95)
Ole e 2	69.8% (0.89 ± 0.79)
Ole e 10	54.1% (0.94 ± 0.79)

This table has been modified from a previously published paper [22].

**Table 3:** TNF-*α*, IL-10, and TGF-*β* genotype and allele frequencies.

Polymorphism	Genotypes and alleles	Patients (*n* = 146)	Controls (*n* = 50)	Asthma (*n* = 109)	Rhinitis (*n* = 37)	*P* value	
		*n* (%)	*n* (%)	*n* (%)	*n* (%)	Controls versus patients	Asthma versus rhinitis
*TNFA-308G>A *	GG	116 (79.4)	40 (80)	85 (78)	31 (83.8)	ns	ns
	GA	28 (19.2)	9 (18)	22 (20.2)	6 (16.2)	ns	ns
	AA	2 (1.4)	1 (2)	2 (1.8)	0	ns	ns
	G	260 (89%)	89 (89%)	192 (88%)	68 (91.8%)	ns	ns
	A	32 (11%)	11 (11%)	26 (12%)	6 (8.2%)	ns	ns

*TNFA-857C>T *	CC	122 (83.6)	44 (88)	92 (84.4)	30 (81.1)	ns	ns
	CT	21 (14.4)	6 (12)	15 (13.8)	6 (16.2)	ns	ns
	TT	3 (2)	0	2 (1.8)	1 (2.7)	ns	ns
	C	265 (90.7%)	94 (94%)	199 (91.2%)	66 (89.2)	ns	ns
	T	27 (9.3%)	6 (6%)	19 (8.8%)	8 (10.8)	ns	ns

*TNFA-1031C>T *	CC	7 (4.8)	6 (12)	6 (5.5)	1 (2.7)	ns	ns
	CT	59 (40.4)	21 (42)	44 (40.4)	15 (40.5)	ns	ns
	TT	80 (54.8)	23 (46)	59 (54.1)	21 (56.8)	ns	ns
	C	73 (25%)	33 (33%)	56 (25.6%)	17 (23%)	ns	ns
	T	219 (75%)	67 (67%)	162 (74.4%)	57 (77%)	ns	ns

*IL-10-571C>A *	CC	82 (56.2)	27 (54)	62 (56.8)	20 (54.1)	ns	ns
	CA	58 (39.7)	18 (36)	43 (39.4)	15 (40.5)	ns	ns
	AA	6 (4.1)	5 (10)	4 (3.6)	2 (5.41)	ns	ns
	C	222 (76%)	72 (72%)	167 (76.6%)	55 (74.3%)	ns	ns
	A	70 (24%)	28 (28%)	51 (23.4%)	19 (25.7%)	ns	ns

*IL-10-1117A>G *	AA	49 (33.5)	21 (42)	39 (35.7)	10 (27)	ns	ns
	AG	62 (42.4)	22 (44)	42 (38.5)	20 (54)	ns	ns
	GG	35 (23.9)	7 (14)	28 (25.6)	7 (18.9)	ns	ns
	A	160 (54.8%)	64 (64%)	120 (55%)	40 (54%)	ns	ns
	G	132 (45.2%)	36 (36%)	98 (45%)	34 (46%)	ns	ns

*TGF-*β*-C-509-T *	CC	56 (38.3)	26 (52)	44 (40.4)	12 (32.4)	ns	ns
	**CT**	**83 (56.8)**	**20 (40)**	60 (55)	23 (62.1)	**P** *** *=* *0.039** **0R* *=* *1.97** ** (95% CI 1.02–3.8)**	ns
	TT	7 (4.7)	4 (8)	5 (4.6)	2 (5.4)	ns	ns
	C	195 (66.7%)	72 (72%)	148 (67.8%)	47 (63.5%)	ns	ns
	T	97 (33.3%)	28 (28%)	70 (32.2%)	27 (36.5%)	ns	ns

**Table 4:** Relevant results of TNF-*α* and IL-10 polymorphisms according to Ole e 2 and Ole e 10 sensitization.

Polymorphism	Genotypes	IL-10 levels (pg/mL)	Ole e 2 sensitized (*n* = 102)	Ole e 2 nonsensitized (*n* = 44)	Ole e 2 asthma (*n* = 81)	Asthma Ole e 2 nonsensitized (*n* = 28)	*P* value	
			*n* (%)	*n* (%)	*n* (%)	*n* (%)	Ole e 2 sensitized versus Ole e 2 nonsensitized	Ole e 2 asthma versus non-Ole e 2 asthma
IL-10-571C>A	CC	7.77 ± 5.4	51 (50)	31 (70.5)	40 (49.4)	22 (78.6)	*P* = 0.029 OR: 0.41 (95% CI 0.19–0.8)	*P* = 0.008 OR: 0.26 (95% CI 0.09–0.7)
	CA	8.76 ± 5.3	45 (44.1)	13 (29.5)	37 (45.7)	6 (21.4)	ns	*P* = 0.02 OR: 3.08 (95% CI: 1.13–8.4)
	AA	5.9 ± 6.1	6 (5.9)	0	4 (4.9)	0	ns	ns

Polymorphism	Genotypes	TNF-*α* levels (pg/mL)	Ole e 10 sensitized (*n* = 79)	Ole e 10 nonsensitized (*n* = 67)	Ole e 10 asthma (*n* = 66)	Asthma Ole e 10 nonsensitized (*n* = 43)	*P* value	
			*n* (%)	*n* (%)	*n* (%)	*n* (%)	Ole e 10 sensitized versus Ole e 10 nonsensitized	Ole e 10 asthma versus non-Ole e 10 asthma

TNFA-308G>A	GG	7.01 ± 4.8	59 (74.7)	57 (85.1)	49 (74.2)	36 (83.7)	ns	ns
	GA	7.79 ± 4.5	20 (25.3)	8 (11.9)	17 (25.8)	5 (11.6)	*P* = 0.04 OR: 2.5 (95% CI 1.02–6.1)	ns
	AA	9.3 ± 6.1	0	2 (3)	0	2 (4.7)	ns	ns

Data show only significant results (*P* < 0.05).
